# Estimation of water retention parameters from nuclear magnetic resonance relaxation time distributions

**DOI:** 10.1002/wrcr.20207

**Published:** 2013-04-23

**Authors:** Stephan Costabel, Ugur Yaramanci

**Affiliations:** 1 Department of Groundwater and Soil Sciences, Federal Institute for Geosciences and Natural ResourcesBerlin, Germany; 2 Department of Applied Geophysics, Berlin University of TechnologyBerlin, Germany; 3 Leibniz Institute for Applied GeophysicsHannover, Germany

**Keywords:** nuclear magnetic resonance, NMR relaxometry, water retention curve, pore size distribution, pore constrictions, sand-clay mixtures

## Abstract

[1] For characterizing water flow in the vadose zone, the water retention curve (WRC) of the soil must be known. Because conventional WRC measurements demand much time and effort in the laboratory, alternative methods with shortened measurement duration are desired. The WRC can be estimated, for instance, from the cumulative pore size distribution (PSD) of the investigated material. Geophysical applications of nuclear magnetic resonance (NMR) relaxometry have successfully been applied to recover PSDs of sandstones and limestones. It is therefore expected that the multiexponential analysis of the NMR signal from water-saturated loose sediments leads to a reliable estimation of the WRC. We propose an approach to estimate the WRC using the cumulative NMR relaxation time distribution and approximate it with the well-known van-Genuchten (VG) model. Thereby, the VG parameter *n*, which controls the curvature of the WRC, is of particular interest, because it is the essential parameter to predict the relative hydraulic conductivity. The NMR curves are calibrated with only two conventional WRC measurements, first, to determine the residual water content and, second, to define a fixed point that relates the relaxation time to a corresponding capillary pressure. We test our approach with natural and artificial soil samples and compare the NMR-based results to WRC measurements using a pressure plate apparatus and to WRC predictions from the software ROSETTA. We found that for sandy soils *n* can reliably be estimated with NMR, whereas for samples with clay and silt contents higher than 10% the estimation fails. This is the case when the hydraulic properties of the soil are mainly controlled by the pore constrictions. For such samples, the sensitivity of the NMR method for the pore bodies hampers a plausible WRC estimation.

**Citation:** Costabel, S., and U. Yaramanci (2013), Estimation of water retention parameters from nuclear magnetic resonance relaxation time distributions, Water Resour. Res., 49, 2068-2079, doi:10.1002/wrcr.20207.

## 1. Introduction

[2] Geophysical applications of nuclear magnetic resonance (NMR) provide, on the one hand, the opportunity to measure the water content of soils and rocks directly. On the other hand, they provide useful information about the pore space properties. NMR applications are available on the laboratory and on the field scale, respectively [e.g., *Coates et al*., 1999; *Dunn et al*., 2002; *Mohnke and Yaramanci*, 2008; *Yaramanci and Mueller-Petke*, 2009]. For saturated material, NMR relaxation data are related to the pore size distribution (PSD), which allows for estimating the saturated hydraulic conductivity, given that the underlying empirical equations can be calibrated adequately [*Mohnke and Yaramanci*, 2008]. It is well known that a multiexponential representation of NMR relaxation data measured in consolidated material in the laboratory and in boreholes can be correlated with capillary pressure curves [e.g., *Kleinberg*, 1996; *Kenyon*, 1997; *Volokitin et al*., 2001].

[3] The NMR relaxation behavior encodes relevant information about the water filled pore space, even at partial saturation [*Bird et al*., 2005; *Hertzog et al*., 2007]. On the laboratory scale, as well as on the field scale, NMR techniques are expected to have great potential for characterizing the vadose zone [*Roy and Lubczynski*, 2005; *Costabel and Yaramanci*, 2011a]. *Chen et al*. [1994] proposed an approach to estimate the relative hydraulic conductivity from mean NMR relaxation times at partial saturation. This approach was verified for glass beads by *Ioannidis et al*. [2006] and for sand of different grain sizes by *Costabel and Yaramanci* [2011b].

[4] The key for soil physical vadose zone investigations is the soil water retention curve (WRC) [*Kutilek and Nielson*, 1994; *Warrick*, 2003]. *Jaeger et al*. [2009] attempted to estimate PSDs of soil samples from NMR by means of pore diameters and compared them to WRC measurements for the first time. They found that a correlation of both can only be established by a complicated and time consuming calibrating process. They concluded that the advantage of NMR to provide fast and time-saving WRC estimates cannot be taken. However, in this study we propose an alternative approach that avoids a quantitative estimation of the PSD and, in doing so, reduces the risk of systematic errors. Instead, we adapt the geophysical interpretation scheme for consolidated rocks that is usually applied to NMR well logging data with focus on oil prospection [*Coates et al*., 1999]. Using this approach, only two additional water retention measurements have to be conducted in addition to the NMR measurement at saturation for estimating the WRC from the NMR relaxation time distribution (RTD). We investigate and assess its potential with artificial and natural soil samples.

## 2. Water Retention Curve and Pore Size Distribution

[5] The WRC describes the relationship of the volumetric water content *θ* in a pore system and its matric potential, i.e., the potential of the pore space to retain the water by capillary forces. The capillary pressure head *h* is a suitable measure of the matric potential expressed as energy per unit weight, i.e., as units of length [*Warrick*, 2003; *Kutilek and Nielson*, 1994]. In contrast to the hydraulic pressure head below the water table, the capillary pressure head is defined negatively to account for the suction effect of the capillary forces against the gravity force. The soil pore space is usually assumed to be a bundle of capillary tubes. When considering a single capillary with circular cross-section, its radius *r* and *h* are related to each other. The smaller *r* is, the higher the potential is for the capillary tube to retain the water. This relationship is given by:


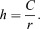
(1)

[6] The proportionality parameter *C* accounts for the physical properties of the interface between the wetting fluid and the pore surface, such as the surface tension, contact angle, and fluid density. With equation (1), the WRC, that is, the function *θ*(*h*) can be considered to be a cumulative pore radius distribution *θ*(*r*) [*Kutilek and Nielson*, 1994]. Considering the capillary bundle model, a point on the WRC is associated with the effective radius *r*_eff_ [*Warrick*, 2003]. At this point, all capillaries with *r < r*_eff_ are filled with water and the capillaries with *r > r*_eff_ are empty.

[7] The actual shape of the WRC depends on various parameters, e.g., grain size distribution, bulk dry density, and contents of clay and organic matter [*Vereecken*, 1995; *Saxton and Rawls*, 2006]. However, a strictly physical formulation of the WRC is very difficult. Thus, in soil physics empirical models are the common choice. An often used model for the WRC is the model of *van Genuchten* [1980], hereafter referred to as the VG model:


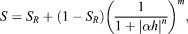
(2)

[8] The saturation degree *S* is the ratio of *θ* and the water content at saturation *θ_S_*. *S_R_* is the residual saturation degree, which is the proportion of water in the smallest pores whose participation in any water transport processes is negligible. The parameters *α*, *n*, and *m* are empirical parameters that are found in practice by fitting WRC measurements. In the most cases, the relationship *m=*1−1/*n* is used, which is referred to as the constrained form [*Peters and Durner*, 2006].

## 3. NMR Relaxation and Pore Size Distribution

[9] Geophysical NMR methods are based on the fact that the ^1^H protons in water molecules have a magnetic dipole moment caused by their nuclear spin angular momentum. In a static magnetic field *B*_0_, the nuclear spins align with *B*_0_, which results in an additional macroscopic magnetization *M* orientated in the same direction as *B*_0_. Conventional NMR methods in the laboratory and in boreholes use artificial *B*_0_ fields with strengths of about 0.050 up to 100 Tesla. The magnitude of *M* at equilibrium is proportional to the number of ^1^H protons in the observed volume, i.e., to the water volume in the sample.The energizing magnetic field *B*_1_ oscillates exactly at the precession frequency of the spins (Larmor frequency) and forces *M* to tilt away from its equilibrium position. After terminating *B*_1_, *M* precesses at the Larmor frequency around *B*_0_ and relaxes back to its state of equilibrium due to an energy exchange between the nuclear spins and their environment. The relaxation of *M* is measured and analyzed. In doing so, two kinds of relaxations are observed, the longitudinal or *T*_1_ relaxation movement parallel to *B*_0_ (*z* direction) and the transverse or *T*_2_ relaxation perpendicular to *B*_0_ (*xy* plane). A detailed description of the underlying physical phenomenon and geophysical measurement principles can be found for instance in *Coates et al*. [1999] and *Dunn et al*. [2002].

[10] The relaxing NMR signal in porous media is, in general, a multiexponentially decaying signal. In case of observing the longitudinal relaxation, the detected NMR signal is given by


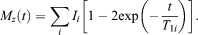
(3)

[11] When observing the transverse relaxation, one detects a signal according to


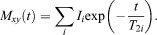
(4)

[12] In equations (3) and (4), *I_i_* is the portion of the signal relaxing with *T*_1_*_i_* and *T*_2_*_i_*, respectively. The initial signal amplitude *M*(*t=*0)*=M*_0_ in both cases linearly depends on the amount of protons in the entire pore space, i.e., *M*_0_ is proportional to *θ*:


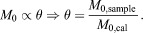
(5)

[13] Consequently, *θ* can simply be determined for a sample from its initial NMR signal amplitude *M*_0_*_,_*_sample_ by calibration, e.g., with a measurement of pure water yielding the reference signal amplitude *M*_0_,_cal_. Laboratory and borehole NMR measurements usually provide the water content with very high accuracy [*Coates et al*., 1999; *Dunn et al*., 2002]. For partially saturated samples the saturation degree *S* can be determined from *M*_0_ by


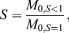
(6)

where *M*_0_*_,S<_*_1_ and *M*_0_*_,S=_*_1_ are the initial amplitudes of the unsaturated and the saturated sample, respectively.

[14] To analyze the basic relaxation processes in a porous medium, let us consider at first the relaxation in a single pore. The *T*_1_ relaxation rate of a water saturated pore consists of two components:


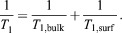
(7)

[15] *T*_1_*_,_*_bulk_ is the bulk fluid relaxation time, i.e., the relaxation time of the pure pore fluid, and *T*_1_*_,_*_surf_ is the surface relaxation time that describes the influence of the pore walls. In the most cases, *T*_1_*_,_*_bulk_ is long compared to *T*_1_*_,_*_surf_, and is therefore often neglected. In general, when observing the *T*_2_ relaxation, an additional relaxation component must be considered in addition to the bulk and the surface relaxation times (*T*_2_*_,_*_bulk_ and *T*_2_*_,_*_surf_):


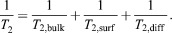
(8)

[16] The diffusion relaxation rate 1/*T*_2_*_,_*_diff_ is nonzero when **B_0_** is inhomogeneous and the spins diffuse through this gradient field during the relaxation process. However, *Keating and Knight* [2007, 2008] have shown that 1/*T*_2_*_,_*_diff_ must be taken into account only in cases where the material to be investigated contains minerals with high magnetic susceptibilities. In our study, both the bulk and the diffusion relaxation rates are neglected.

[17] The surface relaxation rate for both kinds of relaxation accounts for the interaction of the relaxing spins and the pore surface, and is described by


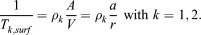
(9)

[18] The ratio A/V is the pore surface-to-volume ratio and *ρ_k_* (*k* = 1, 2) is a material constant, the surface relaxivity, characterizing the influence of paramagnetic impurities at the pore surface [*Foley et al*., 1996].The ratio *A/V* is proportional to the reciprocal pore radius *r*. The geometry factor *a* depends on the pore shape, e.g., *a* = 1 for planar, *a* = 2 for cylindrical, and *a* = 3 for spherical pore geometry. Strictly speaking, equation (9) is only true in the case of fast diffusion, i.e., the diffusion movement of the spins through the pore is fast enough, so that all spins are affected by the pore surface more or less equally [*Brownstein and Tarr*, 1979]. In other words, every spin relaxes back to equilibrium due to the exchange of its energy with the pore wall. In contrast, in the slow diffusion regime a significant proportion of the spins exchange their energy only with their neighboring spins, i.e., they do not “realize” the existence of the pore wall. This is the case, when the material has relatively big pores (e.g., coarse sand and gravel) combined with a high *ρ_k_* [*Brownstein and Tarr*, 1979]. However, the summary of various investigations in, for instance, *Dunn et al*. [2002] suggests that the assumption of the fast diffusion regime is often justified, at least, when investigating natural rocks. For this study, we also assume the fast diffusion regime for both the natural and the artificial samples, because the grain size of the investigated material is relatively small (medium to fine sand, silt, and clay) and impurities with high-magnetic susceptibilities are not expected.Therefore, we can take advantage of the proportionality between *T_k__,_*_surf_ and *r* (equation (9)):


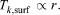
(10)

[19] For the purpose discussed in this paper, we also take advantage of the relationship between *r* and *h* (see equation (2)). Assuming again that the observed pore is a capillary tube, we can relate *T_k__,_*_surf_ to the reciprocal capillary pressure head:


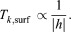
(11)

[20] To interpret an NMR signal in terms of its PSD, it is necessary to reconstruct its multiexponential behavior. This is usually done using an inverse Laplace transform algorithm to invert equations (3) and (4) [*Dunn et al*., 2002]. We show the underlying principle here only for the transverse relaxation. However, the adaptation of the inversion algorithms for the longitudinal relaxation is analog to the following description. One might change the notation of equation (4) to account for the discrete time sampling, i.e., the detected signal *M_j_* at time *t_j_* is given by:


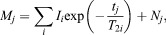
(12)

where *N_j_* is the measurement noise that superposes the NMR response signal. For inverting this equation to get *I_i_* as a function of *T*_2_*_i_*, i.e., the RTD, a minimizing problem has to be solved by means of least squares. Due to the ill-posed character of this problem, the inversion calculation has to be regularized. This regularization is realized by including smoothness constraints to account for the fact that geologically relevant porous material naturally exhibits statistically distributed pores of different sizes. Various strategies for including smoothness constraints can be applied [*Dunn et al*., 2002]. We use the so-called norm smoothing:


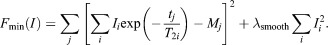
(13)

[21] The parameter *λ*_smooth_ controls the weighting of the smoothness constraints. It is chosen considering the actual noise level of the data. When *λ*_smooth_ is too small, the problem is under-regularized and the noise will be misinterpreted as a part of the model. With increasing *λ*_smooth_, the smoothness of the model increases until the data approximation suffers and the result gets more and more implausible. The usual trade-off is the convention-like assumption that the optimal distribution of relaxation times shows the highest degree of smoothness within the plausibility range given by the noise level of the measurement.

**Figure 1:**
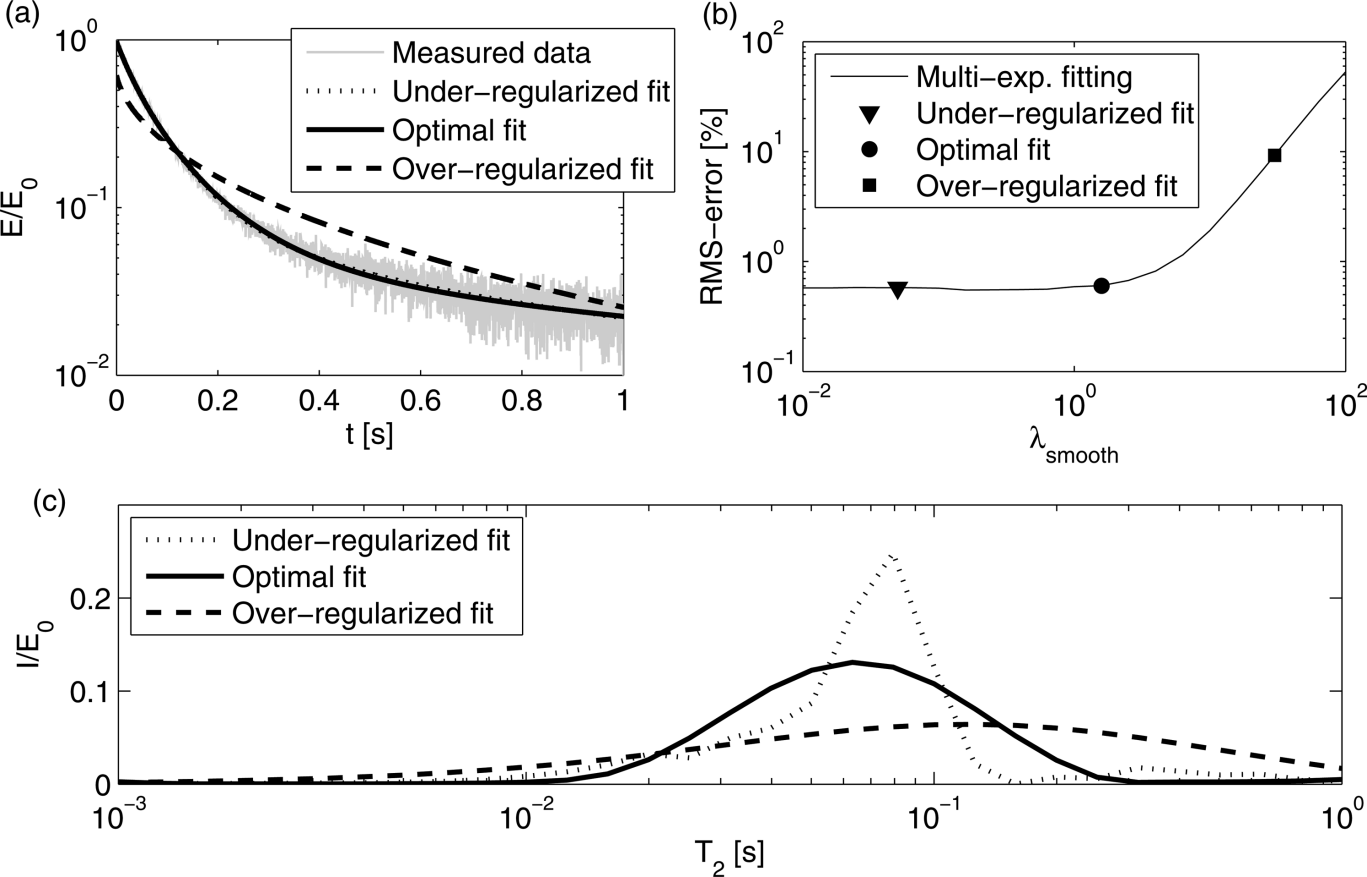
A data example demonstrates the principle of the multiexponential approximation of the NMR signal using the smoothed inverse Laplace transform: (a) the original data and the approximations for different smoothness constraints, (b) the root mean square (RMS) error of various data approximations versus *λ*_smooth_, that is, the parameter controlling the smoothness constraints, and (c) the resulting RTDs for the three examples highlighted in Figure 1b with symbols.

[22] The principle of this data approximation is depicted in Figure 1. Figure 1a shows a data example with a *T*_2_ relaxation measurement and three corresponding multiexponential fits for different *λ*_smooth_. In Figure 1b, the relative root mean square (RMS) error for a set of further smooth inversion results is plotted against *λ*_smooth_. Three approximations from this set are highlighted with symbols and the corresponding inverted RTDs are plotted in Figure 1c. The asymptotic value for decreasing *λ*_smooth_ in Figure 1b represents the relative noise level of the data. In this range, the data approximation is always plausible. However, the risk of misinterpretations and inversion artifacts caused by noise features appear, i.e., the inversion is under-regularized. When *λ*_smooth_ increases, the resulting RTD gets smoother, whereby the quality of the data approximation remains nearly the same. The optimal result is found at the largest *λ*_smooth_ that still leads to a data fit of best quality. A further increase of *λ*_smooth_ leads to implausible data approximations represented by increasing RMS errors. The inversion is overregularized in this range. Often, the *λ*_smooth_ value corresponding to the highest curvature of the RMS-versus-*λ*_smooth_ curve is chosen to identify the optimal multiexponential approximation to the data automatically. However, in this work the optimal *λ*_smooth_ is chosen by determining manually the largest *λ*_smooth_ representing the asymptotic RMS value (black circle in Figure 1b).

## 4. Materials and Methods

### 4.1. Combined NMR and WRC Measurements

**Figure 2:**
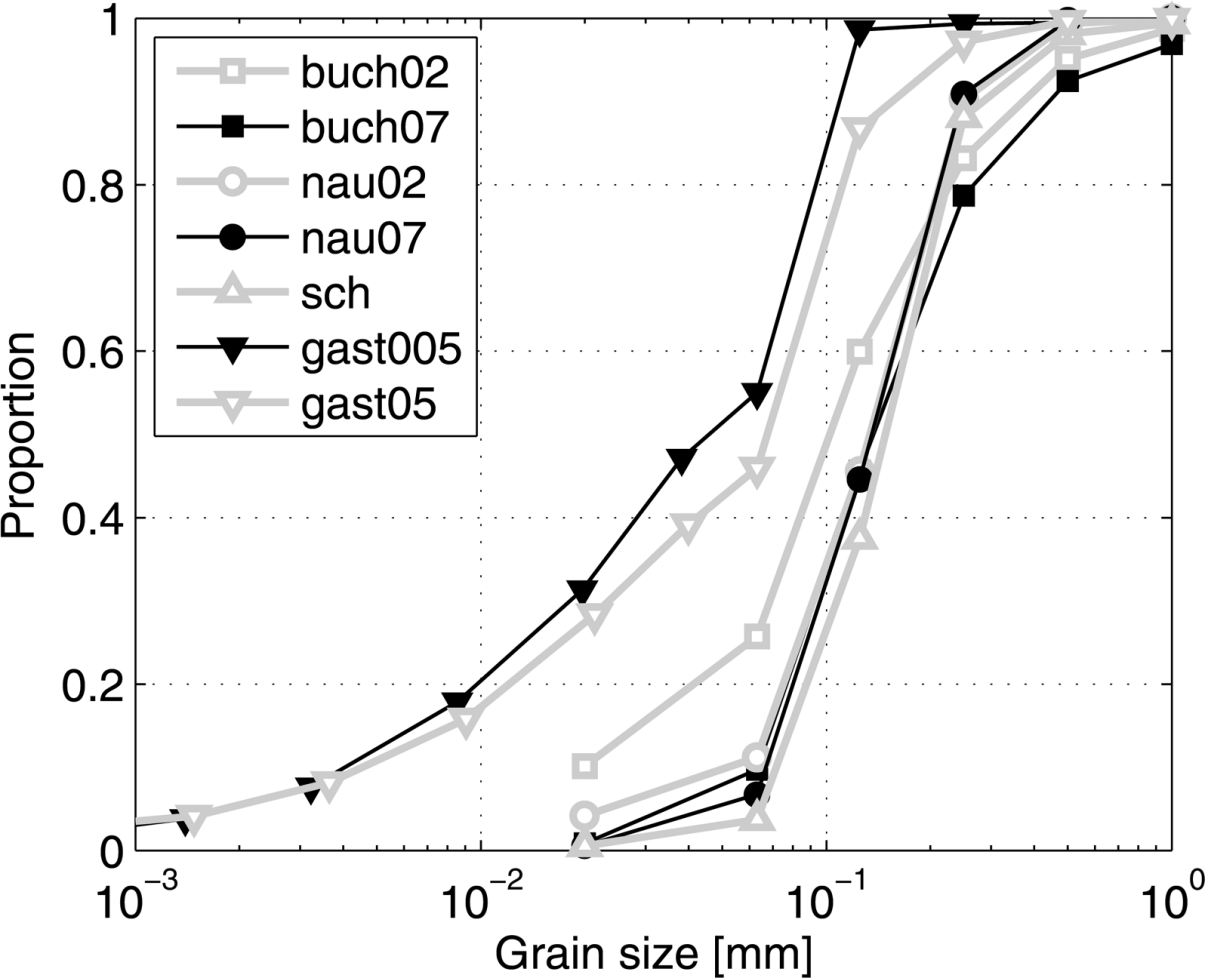
Grain size distributions of the natural soil samples.

[23] Seven natural soil samples were taken from different test locations and depths. The textural and soil physical specifications of the samples are listed in Table 1. Figure 2 shows the cumulative grain size distributions. The test sites Nauen and Buch are located near Berlin, Germany. Schillerlage is in the north of Hannover, Germany. The soil samples from these locations are characteristic for the northern Germany, and consist mainly of fluvial Quaternary sand. The location Gasthof is located in Saxonia, Germany, where the geology is characterized by aeolian Quaternary sediments (loess), i.e., loamy soils with high clay and silt contents.

**Table 1:** Textural Properties of the Natural Soil Samples for the Combined NMR and WRC Measurements: Contents of Clay, Silt, and Sand, BD, and *θ* at *h* = −330 hPa (TH33)

Sample	Location	Depth (m)	Clay (%)	Silt (%)	Sand (%)	BD (g/cm^3^)	TH33 (%)
nau02	Nauen	0.2	0	11	89	1.39	13.59
nau07	Nauen	0.7	0	7	93	1.73	4.14
buch02	Buch	0.2	0	26	74	1.71	18.28
buch07	Buch	0.7	0	10	90	1.67	4.96
sch05	Schillerslage	0.5	0	4	96	1.64	3.65
gast005	Gasthof	0.05	8	79	13	1.47	31.75
gast05	Gasthof	0.5	8	62	30	1.51	28.48

[24] This study aims at the direct comparison of NMR and common WRC measurements using a pressure plate apparatus [*Warrick*, 2003]. Thus, the samples were prepared such that both methods could be applied to the same sample with the same material. In this way, systematic errors resulting from variations of the bulk density (BD, i.e., porosity) of different samples with the same material are avoided. The sample holders were made of polycarbonate tubes of 3.6 cm diameter with a height of 5.5 cm fitting the dimension of the sample inlet of the Maran Ultra NMR apparatus (Resonance Instruments, UK), which was used in this work for the NMR measurements. Filters made of microfiber tissues were mounted at the bottom of the sample holders to realize the hydraulic contact of the sample material and the ceramic plate of the pressure plate apparatus. The samples were saturated with distilled water under vacuum to avoid residual air bubbles during the saturation process. After the initial NMR measurements at saturation, the samples were drained under controlled overpressure according to the conventional pressure plate experiment [*Warrick*, 2003]. During the pressure plate experiments, the top of the sample tubes were open to allow air entry into the pore space, whereas sealing covers at top and bottom during the NMR experiments avoided additional unwanted water losses due to evaporation. The applied pressure for the desaturation of the natural samples ranges from 63 to 2000 hPa in six logarithmically equidistant steps. To reach a state of equilibrium after the application of each pressure step, a waiting period of a few days (about 14–21 days depending on the actual pressure) was necessary until no more water was observed at the outlet of the pressure vessel. After each pressure step, the samples were weighed to determine water content and saturation degree. Afterward, they were sealed to conduct the NMR measurements. Except for the samples from the location Gasthof (see Table 1), this combined measurement scheme could be successfully applied. The two Gasthof samples exhibit water retention properties that could not be investigated with our equipment described above, because the measurement range was limited from 63 to 2000 hPa. To desaturate these samples adequately, a pressure of up to 15,000 hPa was necessary. Therefore, NMR measurements for the Gasthof samples were conducted only at saturation. Afterward, they were sent to an external partner (Department of Ecology at the Berlin University of Technology) for WRC measurements in an extended range from 30 to 15,000 hPa. The extended pressure range was realized by combining measurements with a pressure plate apparatus for higher pressure and a hanging water column [*Warrick*, 2003].

[25] The *T*_1_ measurements were conducted using the inversion recovery sequence with recovery times from 0.1 to 4000 ms. The *T*_2_ relaxation was measured using the CPMG sequence with an interecho spacing time of 150 µs. To achieve an adequate signal-to-noise ratio of the NMR response signals, the stacking rate was initially set to 100 for the measurements of the *T*_2_ relaxation and to 8 for the measurements of the *T*_1_ relaxation (NMR measurements at saturation). During the experiments, the number of stacks was adjusted upward to account for decreasing signals due to decreasing *θ*. Finally, the maximum number of stacks for the *T*_1_ measurements at residual saturation was 49 and for the *T*_2_ measurements 900.

[26] In addition to the natural samples, artificial sand-clay mixtures were prepared to simulate soil samples with decreased pore space. Industrial quartz sand (F32, Quarzwerke GmbH, Germany) was mixed with different portions of kaolinite clay (Kaolin Calypso Pro 83, Caminauer Kaolinwerk GmbH, Germany) from 0 to 100 mass%. Table 2 specifies the clay content (CC), BD and *θ* at *h* = −330 hPa (TH33). For these samples, NMR measurements were conducted only at saturation. The specifications of the NMR measurements are the same as for the natural samples. After conducting the NMR measurements, the samples were sent to the Department of Ecology at the Berlin University of Technology to conduct WRC measurements in the range of 30 to 15,000 hPa. Due to this procedure, the BD of these samples changed and thus, two different BD values for each artificial sample is given in Table 2.

**Table 2:** Specifications of the Sand-Clay Mixtures: CC, BD, *θ* at *h* = −330 hPa (TH33)

CC (mass%)	0	1	3	5	7	10	12	15	20	30	50	70	100
BD, NMR (g/cm^3^)	1.72	1.69	1.66	1.71	1.66	1.65	1.64	1.65	1.57	1.55	1.43	1.26	1.06
BD, WRC (g/cm^3^)	1.47	1.46	1.52	1.55	1.57	1.62	1.63	1.66	1.71	1.83	1.63	1.45	1.26
TH33 (%)	3.07	1.66	3.58	5.29	6.79	10.28	11.69	14.58	18.97	28.87	36.60	44.48	53.71

### 4.2. Estimation of the WRC From NMR

[27] The NMR data were approximated multiexponentially with an inverse Laplace transform algorithm according to the principle described above. This results in a spectral analysis of the NMR relaxation data of the form *I_i_ = f*(*T_i_*), which is referred to as RTD (see also equation (12) and corresponding explanations). For simplification, the notation *T* is used now for both the *T*_2_ and the *T*_1_ relaxation times, whereas the index *i* means the *i*-th relaxation regime, i.e., pore size (see equation (10) and corresponding explanations). The WRC estimation is made using the cumulative RTD of the saturated sample. In doing so, the distribution is normalized with respect to the NMR signal amplitude *M*_0_*_,S=_*_1_ to values between 0 and 1, i.e., to values representing the saturation degree *S* (equation (6)). The cumulative representation of the RTD is then given by


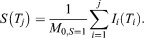
(14)

[28] The WRC is estimated in two steps that are adapted from the usual procedure of estimating the PSD from NMR for sandstones [*Coates*, 1999]. The first step is the determination of *S_R_*. In our study, we considered the water saturation degree at *h* = −2000 cm to be *S_R_*, because this was the highest available pressure step with our experimental equipment. The cumulative RTD is modified such that all relaxation times in the distribution smaller than the cutoff value *T_co_* are associated with a constant *S_R_*


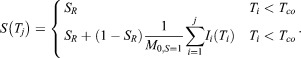
(15)

**Figure 3:**
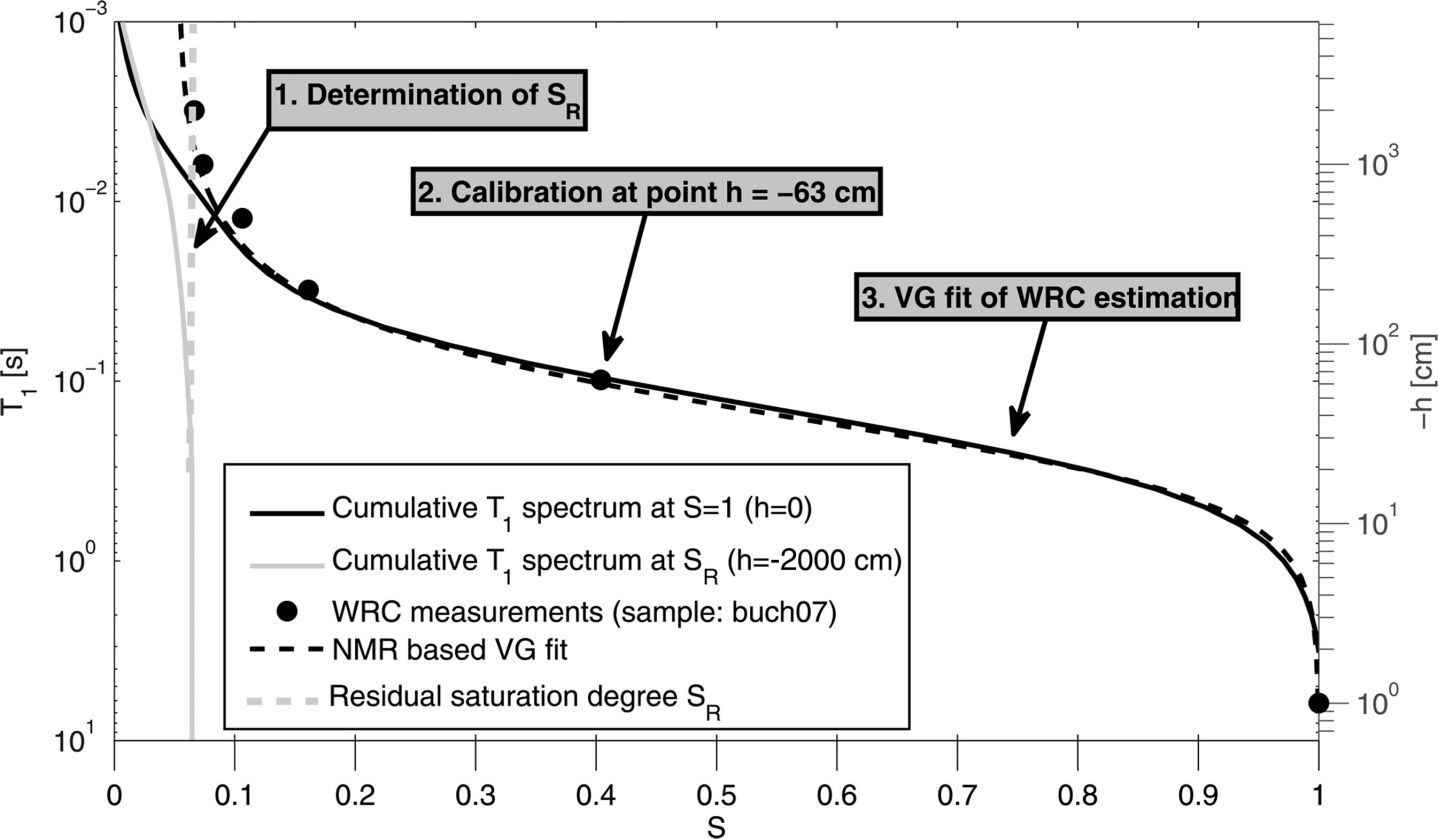
Description of the procedure for estimating the WRC from the cumulative NMR RTD, please see detailed explanations in the text.

[29] In this way, the shape of cumulative RTD becomes very similar to the VG equation (equation (2)) with the asymptotic behavior for increasing *|h|*, i.e., decreasing *r*. The second step is the calibration of the RTD at the base of one certain WRC measurement, e.g., with the pressure plate apparatus. In principle, the pressure head of this measurement can arbitrarily be determined. It just have to be a convenient fixed point somewhere on the WRC, but away from the asymptotic branches for *h* being very high or very low, respectively. We have chosen *h* = −63 cm, because this specific pressure is considered as the beginning of the range in the WRC, at which the remaining pore water is held against the gravity force (field capacity). After determining the calibrating point, the cumulative RTD is shifted at the logarithmic *h*-scale such that it crosses the point *S*(*h* = −63 cm). This shifting is justified by the proportionality between the relaxation time and the reciprocal capillary pressure (see equation (11)), i.e., the shifting factor includes specific material parameters such as the NMR surface relaxivity, as well as the surface tension, the contact angle, and the density of the pore water. The resulting NMR-based WRC estimation is finally fitted using the VG model according to equation (2). In Figure 3, the applied processing steps are depicted with the data of the sample buch07 as an illustrating example.

[30] As a matter of fact, the described calibration procedure for NMR measurements to estimate WRCs includes the determination of *α* and *S_R_*, i.e., the estimation of these parameters from NMR is pointless. However, *n* is the governing VG parameter when estimating the relative hydraulic conductivity as a function of *θ*. An NMR-based estimation of *n* with less effort than using conventional WRC measurements (i.e., only two WRC measurements) would be a great benefit for hydrogeological and soil physical investigations.

### 4.3. WRC Prediction From Texture Information Using the ROSETTA Software

[31] The NMR-based estimates of the VG parameter *n* are compared to the fitting values of real WRC measurements and to parameter predictions from the ROSETTA software, respectively. ROSETTA estimates WRCs from texture information by using a neural network algorithm [*Schaap et al*., 2001]. The calibration of the underlying neural network is based on the UNSODA database [*Leij et al*., 1996]. ROSETTA works with different prediction models that allow different combinations of input parameters. In this study, we use the contents of clay, silt, sand, BD, and the water content at 330 hPa (TH33) as input parameters, which are listed in the Tables 1 and 2.

[32] A reliable VG approximation for the WRC measurements of the natural soil samples conducted in combination with the NMR measurements was not possible, because the resulting WRC database consists of only six data points for each sample in a range too small for an accurate VG fit. As mentioned before, the measurement range of the equipment was limited (63–2000 hPa). Consequently, the NMR-based estimates for the natural soil samples are solely compared to the ROSETTA predictions.

## 5. Results and Discussion

### 5.1. Dependency of T*_1_* and *T_2_* Distributions on Capillary Pressure

**Figure 4:**
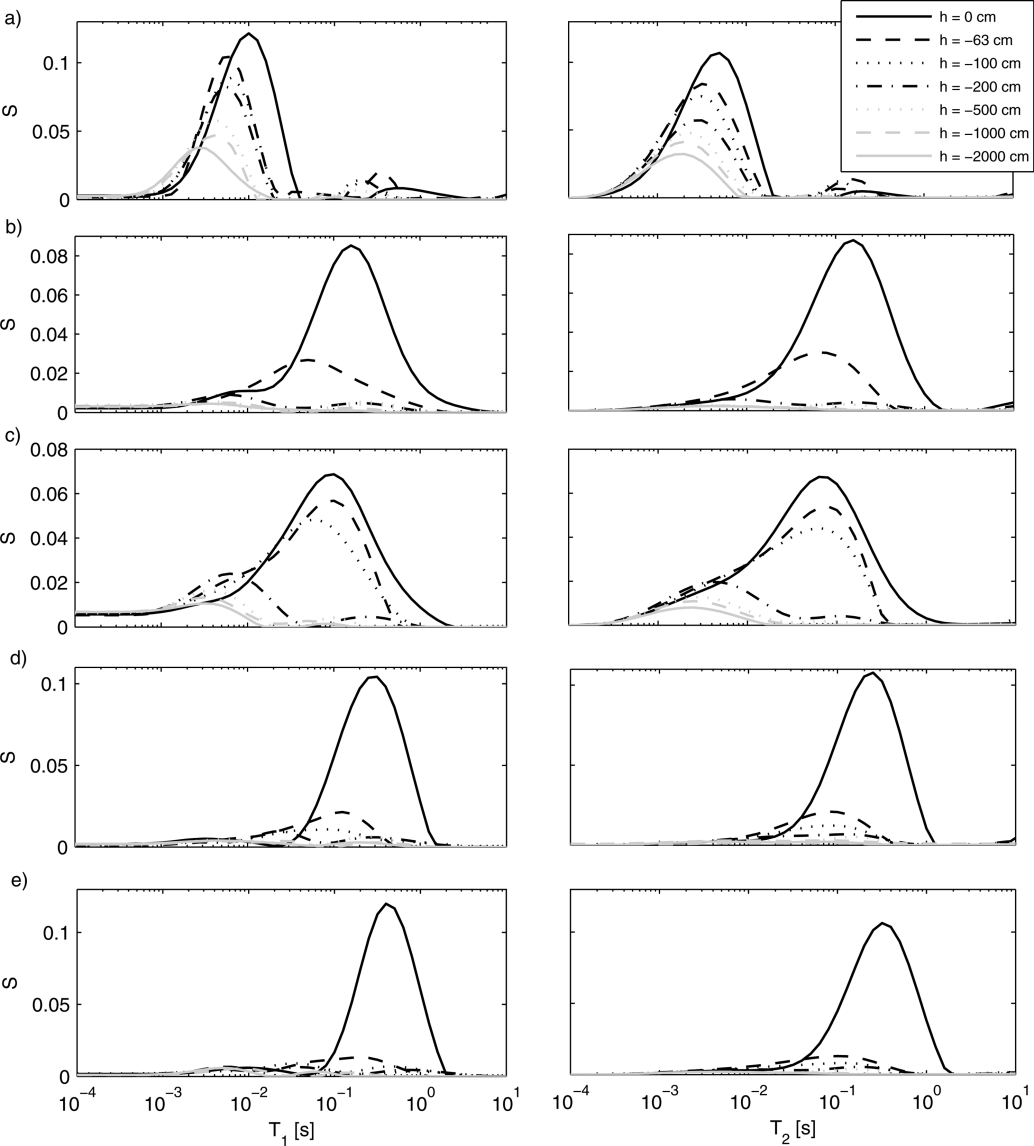
NMR RTDs of natural soil samples at varying capillary pressure heads *h*, *T*_1_ on the left and *T*_2_ on the right-hand side. (a) buch02. (b) buch07. (c) nau02. (d) nau07. (e) sch05.

[33] In Figure 4, *T*_1_ and *T*_2_ distributions are shown for the investigated natural samples. For each sample and relaxation type (i.e., *T*_1_ on the left-hand side and *T*_2_ on the right-hand side of Figure 4), seven distributions are depicted corresponding to the measurement at saturation and six desaturation steps. The distributions are normalized with respect to the initial NMR amplitude *M*_0_ at saturation, i.e., the areas under the RTDs equal the individual saturation degree (see equation (6)).We note that, as expected, the desaturation for all samples starts in the bigger pores related to longer relaxation times, while the remaining water corresponds to smaller relaxation times in the RTDs. We could not observe significant differences between the *T*_1_ and the *T*_2_ relaxation distributions.

[34] The sample buch02 (Figure 4a) shows a small amount of water appearing with decreasing *h* at relaxation times between 0.1 and 1 s, i.e., in bigger pores. This behavior occurs in both the *T*_1_ and the *T*_2_ distributions, so it is quite unlikely that it is due to inversion artifacts. We believe that this observation indicates some kind of redistribution of water after placing the sample from the overpressure regime in the pressure plate apparatus into the NMR device under atmospheric pressure conditions. Similar phenomena are also observed and described by *Bird et al*. [2005], however, the effect is insignificant here and its investigation and discussion is out of the scope of this study. The pore space of sample buch02 is dominated by the smaller pores with relaxation times from 0.001 to 0.03 s that behaves “normally” during desaturation as described above. For the samples nau07 (Figure 4d) and sch05 (Figure 4e), water appears at about 0.02 to 0.05 s in the desaturated *T*_1_ distributions and at about 0.01 to 0.02 s in the desaturated *T*_2_ distributions, whereas the distributions at saturation do not show any water in this range. Similarly, the *T*_1_ distribution of sample nau02 (Figure 4c) shows more water in the range of 0.002–0.01 s during desaturation than under saturated conditions. However, it cannot be decided whether these features are of physical origin, i.e., related to water redistribution inside the pore space [*Bird et al*., 2005], or just inversion artifacts. The *T*_1_ distributions of the sample nau02 show a nonzero asymptotic behavior, which is not reliable from the physical point of view. This feature is certainly an inversion artifact.

### 5.2. WRC Estimation From NMR for the Natural Samples

**Figure 5:**
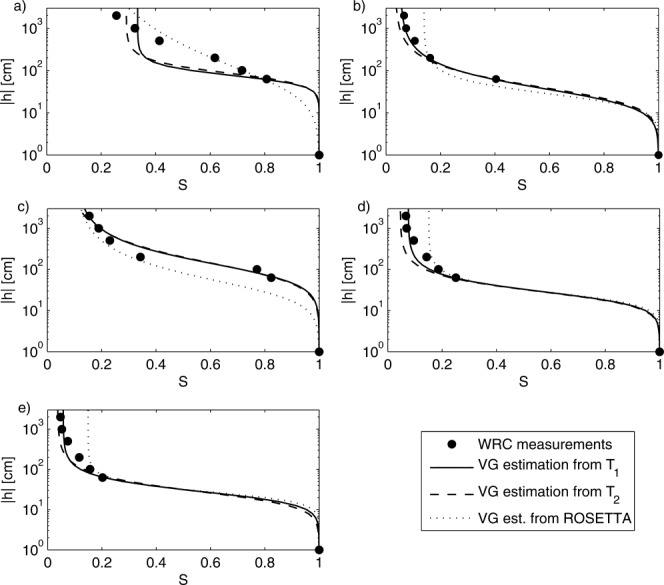
WRC parameterizations after *van Genuchten* [1980] from *T*_1_ and *T*_2_ distributions of natural soil samples compared to real WRC measurements and to predictions from ROSETTA. (a) buch02. (b) buch07. (c) nau02. (d) nau07. (e) sch05.

**Figure 6:**
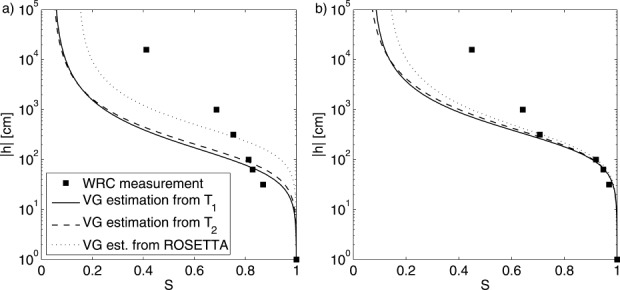
WRC parameterizations after *van Genuchten* [1980] from *T*_1_ and *T*_2_ distributions of natural soil samples from the location Gasthof compared to real WRC measurements and to predictions from ROSETTA. (a) gast005. (b) gast05.

[35] In Figure 5, the NMR-based WRC estimations (i.e., represented by their VG approximations) of the natural samples are compared to the WRC measurements and to the ROSETTA predictions, respectively. For the samples with less silt content and clay content (i.e., samples nau02, nau07, buch07, and sch05, see Table 1), the NMR-based estimations for both the *T*_1_ and the *T*_2_ relaxation measurements are in good agreement with the WRC measurements. Compared to these samples, the sample buch02 has a relatively high content of finer grains (see Figure 2 and Table 1), which is the reason for the shift of the corresponding WRC measurement toward higher *|h|* on the one hand, and the smaller slope of *S* with decreasing *|h|* on the other hand. The NMR estimation fails in this case, the estimated slope of the WRC is much larger than the measurements indicate. The same is true for the samples from the location Gasthof (Figure 6). For the WRC estimations for these two samples, the calibrating procedure for the cumulative RTD was modified slightly. First, *S_R_* could not reliably be determined by the WRC measurements and thus, the *S_R_* values predicted by ROSETTA were used for the calibration. Second, the calibration values for shifting the cumulative NMR distributions were determined from other samples prepared with the same material. Strictly speaking, an unknown systematic error must be expected here due to the fact that different samples even with the same material will likely exhibit different bulk densities. However, this systematic error mainly affects the determination of the VG parameter *α*, and is expected to have a minor effect on the parameter *n* controlling the slope of the WRC [*Haverkamp et al*., 2005]. Thus, comparing the resulting NMR-based WRC estimations with the ROSETTA predictions is nevertheless legitimate.

### 5.3. WRC Estimation From NMR for the Sand-Clay Mixtures

**Figure 7:**
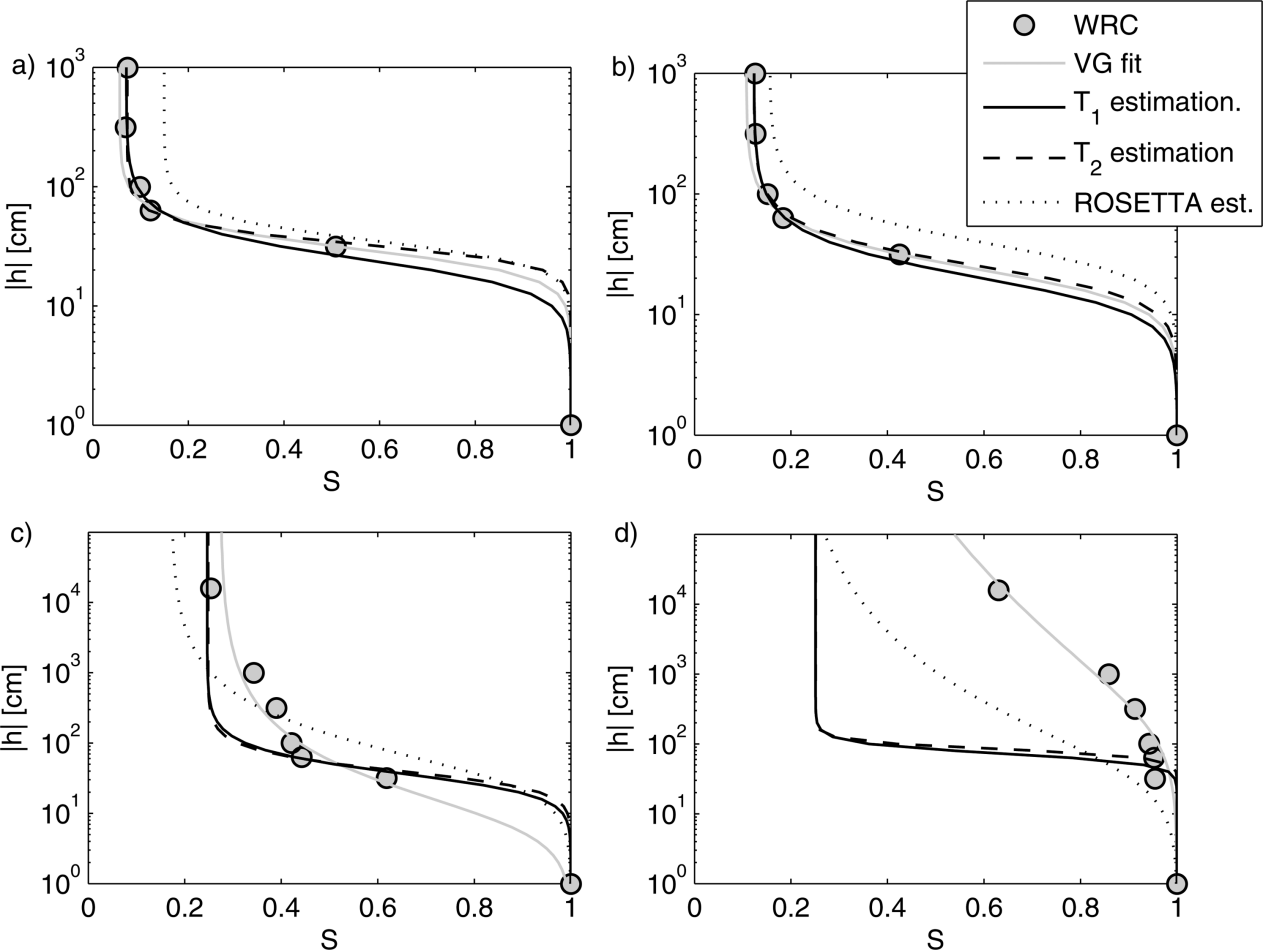
WRC parameterizations after *van Genuchten* [1980] from *T*_1_ and *T*_2_ distributions of artificial sand-clay mixtures compared to real WRC measurements and to predictions from ROSETTA. (a) Sand without clay. (b) Sand with 5% clay. (c) Sand with 15% clay. (d) Sand with 30% clay.

[36] In Figure 7, examples of sand-clay mixtures with CCs of 0, 5, 15, and 30% are shown and compared to the WRC measurements and to the corresponding ROSETTA predictions, respectively. Obviously, the WRC estimations for the samples with CCs smaller than 10% are in good agreement with the measurements, while for higher CC the estimation fails as already observed for the three natural samples described above. Again, the NMR-based WRCs show a systematically overestimated slope of *S* as a function of *|h|*. Moreover, the deviations between real and estimated WRC seem to increase with increasing CC.

**Figure 8:**
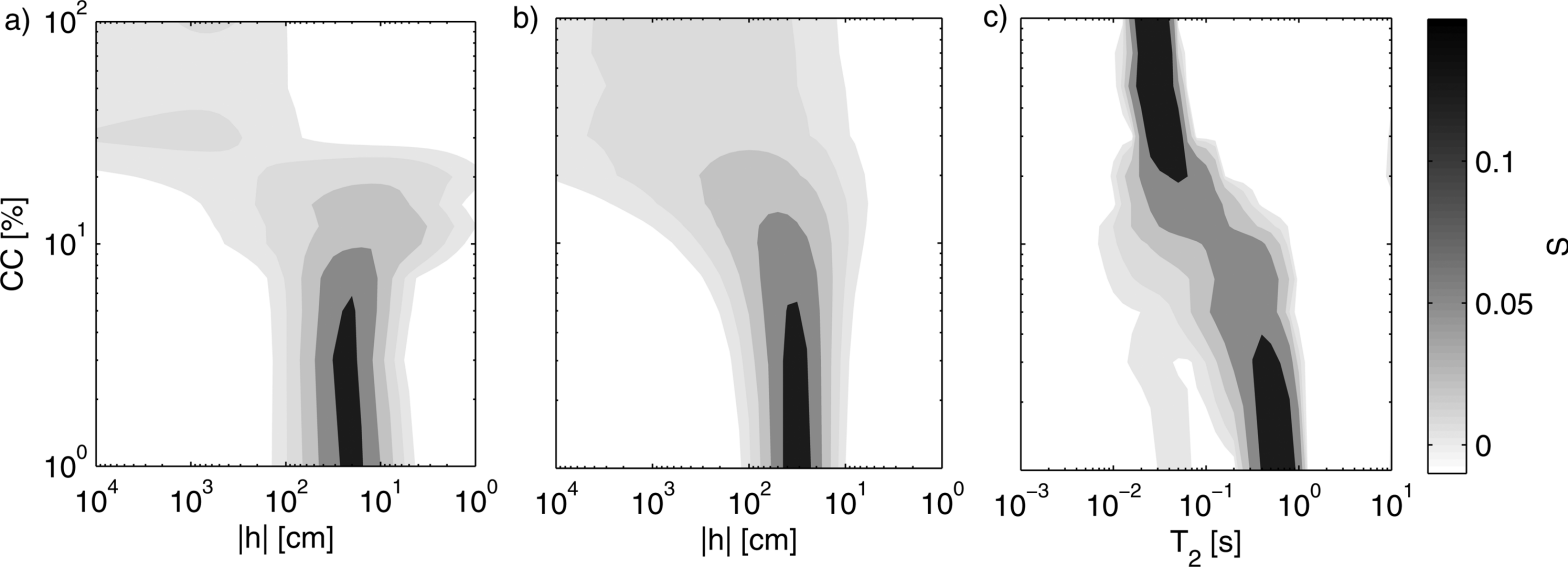
Differentiated WRCs for CCs from 1 to 100% (a) from real WRC measurements and (b) from ROSETTA predictions compared to (c) NMR RTDs.

[37] This effect is shown in Figure 8 more in detail, where the differentiated WRCs derived from the WRC measurements of the sand-clay mixtures, their ROSETTA predictions, and their NMR RTDs at saturation are compared. As mentioned in section 2, assuming the capillary bundle model, the WRC can be considered to be a cumulative PSD. Reciprocally, when differentiating the VG representations of the WRC measurements, we estimate the PSD of the investigated samples. Figure 8a shows the PSD estimations for the sand-clay mixtures depending on the CC in a surface plot. Obviously, the PSD tends to increase with increasing CC. We applied the same procedure to the VG representation of the ROSETTA predictions (Figure 8b) with the same result. As described in section 3, the NMR RTD can also be considered to be a PSD estimation. For comparison, the *T*_2_ distributions are also shown as surface plot in Figure 8c. We note that the *T*_2_ distributions for CCs higher than 10% are much narrower than the differentiated WRCs for the same material. The narrow NMR PSD can be explained by the fact that NMR is much more sensitive to the pore bodies than to the pore throats [*Mohnke and Klitzsch*, 2010]. In contrast to the NMR PSD, WRC measurements under pressure are strongly related to the pore throats: A relatively big pore would hold its water during drainage, if the entry to this pore is too small for the air bubbles at a certain pressure. When the pressure increases the air will enter the pore immediately, while the pore drains almost completely [*Tuller and Or*, 2002]. Consequently, the drainage behavior of the entire pore space in dependency on the applied pressure is dominated completely by the constrictions of the pore space. Obviously, an increasing content of finer material in the pore space of loose sediments leads predominantly to a broadening of the pore throat distribution, whereas the distribution of the pore bodies remains relatively narrow. However, for a CC up to 10% the shape of the *T*_2_ distributions in Figure 8 is quite similar to those of the corresponding differentiated WRCs. In this range, an estimation of the WRC from NMR seems reliable.

[38] In the range of 0.03–0.07 s, the *T*_2_ distributions show small proportions of water. This effect is likely due to the packing of the artificially prepared samples. It is not possible to avoid an accumulation of clay particles during the sample preparation. Consequently, a small amount of water is solely related to the clay particles and shows an isolated peak at small relaxation times in the NMR RTD. However, the impact of these peaks is minor and does not influence the estimation of the VG parameters, because the corresponding amount of water is related to the residual water, anyway.

**Figure 9:**
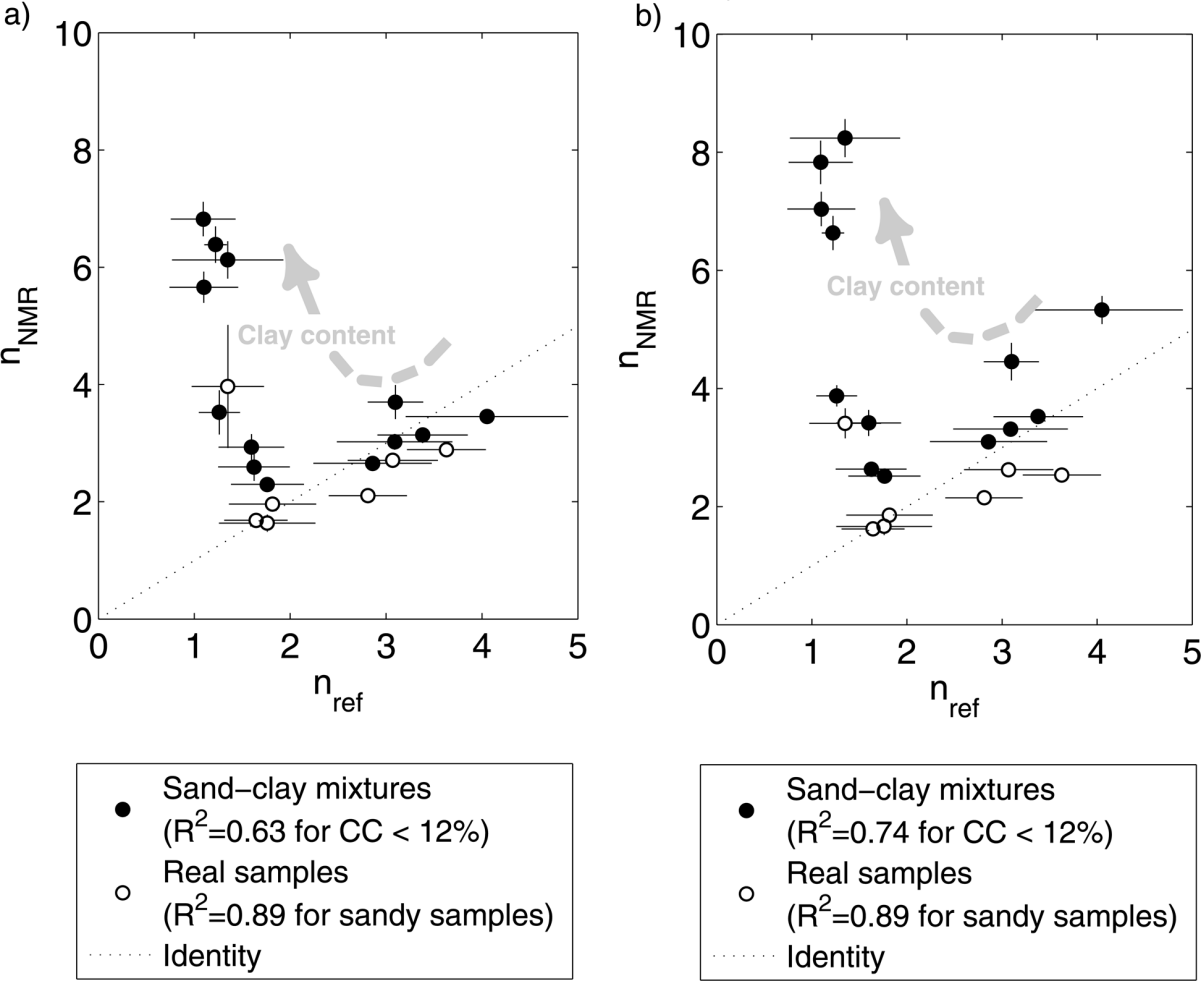
Comparison of the NMR-based estimation of the van Genuchten parameter *n* with reference values for *n*. The reference values for the sand-clay mixtures are calculated from real WRC measurements. For the natural samples, the reference values are predicted by the ROSETTA software. (a) Estimates of *T*_1_ measurements. (b) Estimates of *T*_2_ measurements.

[39] In Figure 9, the NMR-based estimates of the VG parameter *n* are correlated with reference values. The reference values for the sand-clay mixtures are given by the VG approximations of the real WRC measurements and by ROSETTA predictions for the natural soil samples. The error bars in Figure 9 show the 95% uncertainty intervals of the approximations. For the estimations from the *T*_1_ (left-hand side) and the *T*_2_ distributions (right-hand side), the range for plausible *n* estimates starts at approximately at *n* = 2. For smaller values the NMR method tends to overestimate *n*. This overestimation increases with increasing CC up to absolutely unrealistically high values. For samples with such high CCs, all references in the literature report *n* values smaller than 2. The VG parameter *n* can be considered to be a measure for the width of the PSD: Large *n* values indicate narrow PSDs and vice versa. The overestimation of *n* by NMR indicates a strong relation of this method to the pore bodies of the material, whereas the conventional WRC measurements are mainly sensitive to the pore throats.

[40] For clay and silt contents from 0 to approximately 10% the estimation of *n* is plausible. For the natural soil samples, when excluding the silty sand sample buch02, the variance *R^2^* is 0.89 for both the *T*_1_ and the *T*_2_ estimates. The *R^2^* value for the estimates of the sand-clay mixtures is smaller with 0.63 for *T*_1_ and with 0.74 for the *T*_2_ estimates. Please note that, for the sand-clay mixtures, we correlate here the results of different sample sets. For different samples, even with the same material, the BD varies depending on the packing during the sample preparation (see Table 2). Differences in the BD will result in slight variations of the PSD for each sample and, consequently, differing *n* estimates. This leads to the decreased *R^2^* for our data set consisting of the artificial samples compared to the data for the natural soil samples. Future research should focus on the influence of the BD on the NMR relaxation data. When comparing the *T*_1_ and the *T*_2_ results for the sand-clay mixtures, it seems that the *n* estimates from the *T*_2_ measurements tend to be overestimated. This effect should also be investigated in future research. Apart from this, in the reliable range for a plausible estimation of *n*, the uncertainties of the NMR-based estimates ranging from approximately 0.1–0.3 are smaller than both the uncertainties of the real WRC measurements and of the ROSETTA predictions. These uncertainties are much higher with values up to 1.

## 6. Conclusions

[41] In this paper, the potential of NMR relaxometry (*T*_1_ and *T*_2_ relaxation) to estimate WRCs is investigated. An interpretation scheme was suggested that estimates the VG parameters from the cumulative NMR RTDs. This scheme is adapted from the usual method providing the PSDs of consolidated sedimentary rocks with the help of calibration data from mercury injection measurements [*Coates et al*., 1999]. In our approach, the cumulative NMR RTD of a sample at saturation is calibrated by means of two additional WRC measurements: One for determining the residual water saturation *S_R_*, the other one for shifting the RTD on the logarithmic *h*-scale such that it fits a certain fixed point. Afterward, the NMR-based WRC can be approximated with the VG model. The shifting procedure during the calibration is justified by the fact that the NMR relaxation time is proportional to the reciprocal *h*, i.e., the shifting factor includes specific material parameters such as the NMR surface relaxivity and geometrical properties, as well as the surface tension, the contact angle, and the density of the pore water. In this work, all fixed points are determined with the saturation degree of the material at a capillary pressure of *h* = −63 cm. The *S_R_* values for the samples were determined here at a capillary pressure of *h* = −2000 cm.

[42] It could be shown with natural samples and with artificial sand-clay mixtures that the VG parameter *n*, which is the effective parameter for the prediction of the relative hydraulic conductivity as a function of *θ* [*van Genuchten*, 1980], can reliably be estimated for material with clay and silt contents up to 10%. For loose sediments with higher contents of clay and silt, the NMR-based WRC estimation fails. NMR is generally a method with high sensitivity to the pore bodies [*Mohnke and Klitzsch*, 2010], whereas the hydraulic properties represented by the WRC are controlled by the distribution of the pore throats. In loose sediments, a high content of clay or silt leads to a broad distribution of constrictions in the pore space that cannot be recovered by NMR relaxation measurements. In contrast, pore throats and pore bodies in sedimentary rocks have a specific ratio due to the consolidation process [*Coates et al*., 1999; *Dunn et al*., 2002], and the NMR measurement at saturation can reliably provide a proxy also for the pore throat distribution. The findings of this study suggest that in loose sediments and soils this is not the general case. For soils with high contents of clay and silt, the NMR-based PSD measured at saturation is an unsuitable measure for hydraulic processes at partial saturation.

[43] Regarding the WRC approximation of the NMR-based WRCs for sandy soils, the calibrating procedure of our approach makes the approximation of the VG parameters *α* and *S_R_* (see Figure 3 and corresponding explanations) pointless. However, *n* is the governing VG parameter for estimating the relative hydraulic conductivity, so an NMR-based estimation of *n* with only two WRC measurements is a significant benefit for hydrogeological and soil physical investigations, even though this is possible for sandy soils only. Future investigations should establish this method statistically using larger data sets with additional soil samples.

[44] To estimate WRCs of silty and clayey soil samples using NMR relaxometry, future research must consider pore models that account, as alternative to the classical capillary bundle, also for constricting structures in the pore space. To predict the water retention properties of such models, our investigations indicate that NMR relaxometry measurements at saturation cannot provide enough information. Thus, additional methods will be necessary, for instance, measurements of induced polarization (IP) that can be correlated directly with the pore surface [e.g., *Binley et al*., 2005; *Revil*, 2012; *Breede et al*., 2012]. The IP method is therefore expected to be sensitive particularly to the pore throats. Regarding the identified limitations of the approach suggested in this paper, the potential of combining NMR and IP applications should be investigated in the future.

## Key Points

- For sandy soils, water retention parameters can be estimated from NMR data.
- NMR is sensitive for pore bodies only.
- For samples with many pore constrictions, the NMR estimation must fail.
